# Mesenteric lymph node stromal cell‐derived extracellular vesicles contribute to peripheral de novo induction of Foxp3^+^ regulatory T cells

**DOI:** 10.1002/eji.201746960

**Published:** 2017-09-15

**Authors:** Maria Pasztoi, Joern Pezoldt, Michael Beckstette, Christoph Lipps, Dagmar Wirth, Manfred Rohde, Krisztina Paloczi, Edit Iren Buzas, Jochen Huehn

**Affiliations:** ^1^ Experimental Immunology Helmholtz Centre for Infection Research Braunschweig Germany; ^2^ Model Systems for Infection and Immunity Helmholtz Centre for Infection Research Braunschweig Germany; ^3^ Central Facility for Microscopy Helmholtz Centre for Infection Research Braunschweig Germany; ^4^ Department of Genetics, Cell‐ and Immunobiology Semmelweis University Budapest Hungary

**Keywords:** Extracellular vesicles, Fibroblastic reticular cells, Intestinal tolerance, Mesenteric lymph nodes, Regulatory T cells

## Abstract

Intestinal regulatory T cells (Tregs) are fundamental in peripheral tolerance toward commensals and food‐borne antigens. Accordingly, gut‐draining mesenteric lymph nodes (mLNs) represent a site of efficient peripheral de novo Treg induction when compared to skin‐draining peripheral LNs (pLNs), and we had recently shown that LN stromal cells substantially contribute to this process. Here, we aimed to unravel the underlying molecular mechanisms and generated immortalized fibroblastic reticular cell lines (iFRCs) from mLNs and pLNs, allowing unlimited investigation of this rare stromal cell subset. In line with our previous findings, mLN‐iFRCs showed a higher Treg‐inducing capacity when compared to pLN‐iFRCs. RNA‐seq analysis focusing on secreted molecules revealed a more tolerogenic phenotype of mLN‐ as compared to pLN‐iFRCs. Remarkably, mLN‐iFRCs produced substantial numbers of microvesicles (MVs) that carried elevated levels of TGF‐β when compared to pLN‐iFRC‐derived MVs, and these novel players of intercellular communication were shown to be responsible for the tolerogenic properties of mLN‐iFRCs. Thus, stromal cells originating from mLNs contribute to peripheral tolerance by fostering de novo Treg induction using TGF‐β‐carrying MVs. This finding provides novel insights into the subcellular/molecular mechanisms of de novo Treg induction and might serve as promising tool for future therapeutic applications to treat inflammatory disorders.

## Introduction

The gastrointestinal tract harbors the largest number of immune cells throughout the body, and constrains invading pathogens with multiple layers of innate and adaptive immune responses. Simultaneously, the intestinal immune system has to confer tolerance toward self and harmless, non‐self antigens derived from diet and commensals. Microbiota are not only critical for breaking down indigestible nutrients and synthesizing vitamins 1, but also provide stimuli for the development of the mucosal immune system 2, 3, and are especially critical for peripheral de novo induction of Foxp3^+^ regulatory T cells (Tregs) 4, 5. Although the vast majority of Foxp3^+^ Tregs is generated already in the thymus, their T‐cell receptor repertoire is complemented by peripheral de novo Treg induction, allowing establishment of mucosal tolerance 6, 7, 8. De novo Treg induction is more efficient in gut‐draining lymph nodes (LNs), including liver‐draining celiac LN and mesenteric LNs (mLNs), when compared to skin‐draining peripheral LNs (pLNs) 9, 10, 11, 12. Results from our previous study suggest that de novo Treg induction is independent of the antigen administration route 12, but rather determined by intrinsic properties of gut‐draining LNs, including migratory CD103^+^ tolerogenic dendritic cells (DCs) 9, 10, 13 and resident fibroblastic reticular cells (FRCs) located in the T‐cell zone of LNs 12, 14, 15, 16. Both cell types express high levels of retinal aldehyde dehydrogenase 2 (RALDH), thereby favoring de novo Treg induction and fostering expression of gut‐homing molecules on differentiated T cells 9, 10, 13, 14, 15, 17. Moreover, LN transplantation experiments revealed that the unique properties of mLNs are stably imprinted within the stromal cell compartment, as efficient induction of both Foxp3 and gut‐homing molecules was maintained after transplantation of mLN into a non‐tolerogenic skin‐draining site 12, 15.

Since molecular details of the tolerogenic properties of mLN stromal cells are only incompletely understood, we here aimed to identify factors involved in the direct cross‐talk between FRCs and differentiating T cells, and focused on the recently rediscovered way of intercellular communication via extracellular vesicles (EVs). EVs, including small EVs here referred to as exosomes (50–100 nm), intermediate size EVs here referred to as microvesicles (MVs) (100‐1000 nm) and large apoptotic bodies (> 1000 nm in diameter), are involved in various immunological processes 18, 19, 20, 21 and have the potential for both therapeutic and diagnostic applications 22, 23. Release of EVs is a property of almost all cell types 18, 21, 24, 25, including different types of fibroblasts 26, 27. As EVs carry biologically active molecules 25, 26, 28, 29, it was tempting to speculate that secreted molecules of FRCs harboring tolerogenic properties are associated with EVs.

Here, we report that immortalized FRCs (iFRC) originating from gut‐draining mLNs possess a higher Treg‐inducing potential when compared to iFRCs derived from skin‐draining pLN. One of the major factors responsible for these tolerogenic properties of mLN‐iFRCs is TGF‐β, a key cytokine for de novo Treg‐induction. Furthermore, TGF‐β is not simply released into the supernatant (SN), but is associated with MVs. Therefore, these observations reveal the capacity of FRCs in shaping the tolerogenic micromilieu of mLNs via release of immunomodulatory factor‐containing MVs that directly affect T‐cell differentiation, finally resulting in a population of de novo induced Foxp3^+^ Tregs.

## Results

### Immortalized stromal cells derived from mLNs show a high Treg‐inducing capacity

**Figure 1:**
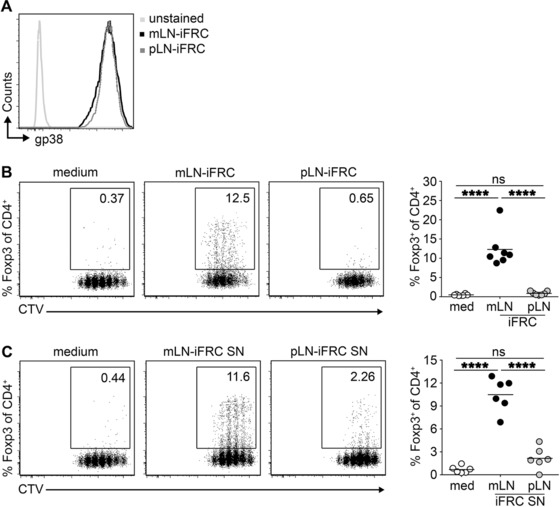
mLN‐iFRCs possess superior de novo Treg‐inducing capacity. (A) FRCs were ex vivo isolated from skin‐draining pLNs and gut‐draining mLNs and immortalized via lentiviral transduction. After doxycycline‐induced expansion, pLN‐ and mLN‐iFRCs were phenotyped by flow cytometry. Representative histograms depict expression of gp38 among CD45^−^CD24^−^CD31^−^ iFRCs originating from mLN and pLN. Histogram is from a single experiment representative of three independent experiments with one mLN‐ and one pLN‐iFRCs per experiment. (B, C) CTV‐labeled naïve CD4^+^ T cells from Foxp3^hCD2^xRag2^‐/‐^xDO11.10 mice were added to iFRCs (B) or iFRC‐derived SNs (C) in presence of IL‐2 and anti‐CD3/CD28 Dynabeads. Four days later, the frequency of de novo induced Foxp3^+^ cells and CD4^+^ T cell proliferation was determined by flow cytometry, as shown in representative dot plots from a single experiment representative of seven (B) or six (C) independent experiments with one mLN‐ and one pLN‐iFRCs per experiment. Numbers in gates indicate frequencies. Each dot within scatterplot depicts mean of technical replicates, and each line represents mean of depicted dots within respective groups. Data were pooled from seven (B) or six (C) independent experiments with one mLN‐ and one pLN‐iFRCs per experiment; *p*‐values were calculated using one‐way ANOVA followed by Bonferroni's post‐test. ns, not significant; ****p < 0.0001. med, medium.

To allow unlimited investigation of LN stromal cells, iFRCs derived from mLNs and pLNs were generated via lentiviral transduction of *ex vivo* isolated FRCs with a doxycycline‐inducible SV40 TAg 30. After in vitro expansion, both mLN‐ and pLN‐iFRCs kept the characteristic CD31^−^gp38^+^ phenotype of FRCs (Fig. 1A), and iFRC proliferation was strictly dependent on doxycycline (data not shown). In order to investigate the direct impact of mLN‐ and pLN‐FRCs on de novo Treg induction, a co‐culture system was established using naïve CD4^+^ T cells and iFRCs in the growth‐arrested state. This system lacks any influence from DCs, but relies on polyclonal T cell stimulation using anti‐CD3/CD28 Dynabeads. In absence of iFRCs, hardly any Foxp3^+^ Tregs were de novo induced from naïve CD4^+^ T cells (Fig. 1B and Supporting Information Fig. 1). However, co‐cultures of naïve CD4^+^ T cells with mLN‐iFRCs resulted in a significantly increased frequency of de novo induced Foxp3^+^ Tregs when compared to co‐cultures with pLN‐iFRCs, in line with the previously described differential Treg‐inducing capacity of ex vivo isolated stromal cells from mLNs and pLNs 12. In order to unravel how iFRCs communicate with T cells and to identify the molecular mechanisms underlying the superior Treg‐inducing properties of mLN‐iFRCs, we next studied the Treg‐inducing capacity of iFRC‐derived SN containing secreted factors and subcellular structures. Interestingly, SN of mLN‐iFRCs was more efficient in supporting de novo Treg induction when compared to pLN‐iFRC‐derived SN (Fig. 1C). These data suggest that FRCs from mLNs can release soluble factors and/or subcellular structures that favor conversion of naïve T cells into Foxp3^+^ Tregs, thereby contributing to the unique tolerogenic microenvironment of mLNs.

### Multiple soluble factors are differentially expressed between mLN‐ and pLN‐iFRCs

**Figure 2:**
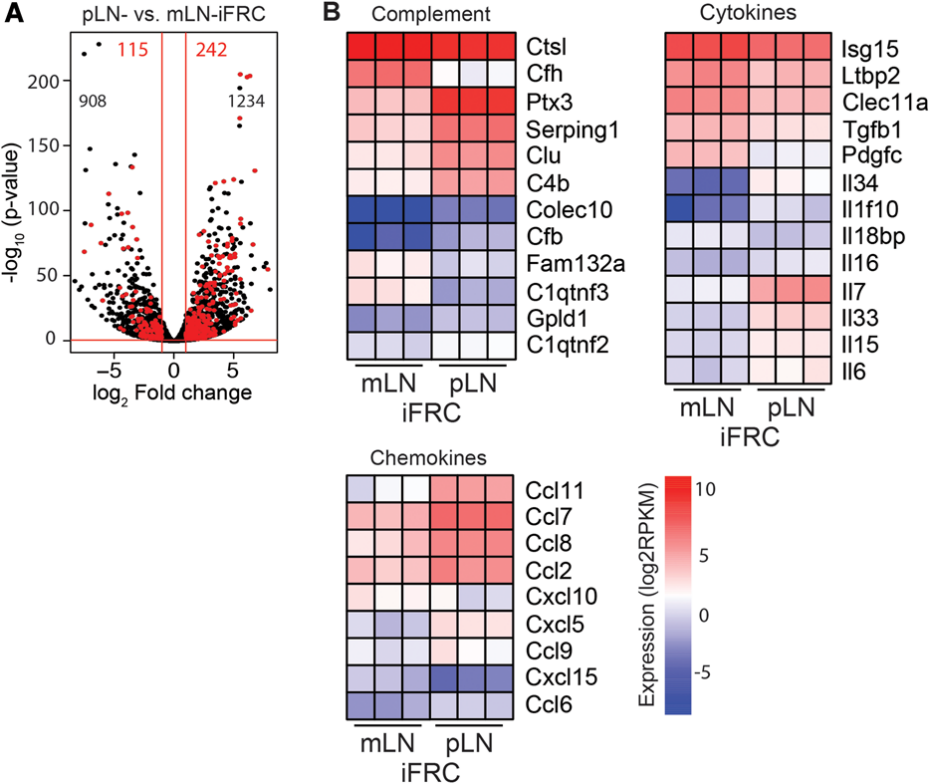
Differentially expressed soluble factors of mLN‐ and pLN‐iFRCs. RNA‐seq analysis was performed on mLN‐ and pLN‐iFRCs. Genes with |log_2_(FC)| ≥ 1 and *q*‐value ≤ 0.05 were considered differentially expressed. (A) Volcano plot represents a total of 14 919 transcripts. Black numbers indicate genes encoding non‐secreted molecules being up‐ (left) and downregulated (right) in mLN‐iFRCs as compared to pLN‐iFRCs. Significantly up‐ and downregulated secreted‐molecule encoding genes of mLN‐iFRCs compared to pLN‐iFRCs are highlighted in red. (B) Heatmaps represent groups of secreted protein‐encoding differentially expressed genes of mLN‐ and pLN‐iFRCs. Color‐coding is based on RPKM normalized count values. Data from a single experiment with cells from three independent cultures of mLN‐ and pLN‐iFRCs are depicted. FC, fold change; RPKM, reads per kilobase maximal transcript length per million mapped reads.

To define secreted molecules responsible for the differential Treg‐inducing capacity of mLN‐ and pLN‐iFRCs, we performed an RNA‐seq analysis. Out of 14919 profiled transcripts, 1023 and 1476 genes were up‐ and downregulated, respectively, in mLN‐iFRCs when compared to pLN‐iFRCs (|log_2_(FC)| ≥ 1, adjusted *p*‐value ≤ 0.05) (Fig. 2A; Supporting Information Table 1). Utilizing *Uniprot* [http://www.uniprot.org/, 22.02.2016, “secreted”] as reference allowed to identify 115 and 242 secreted protein‐encoding genes being up‐ and downregulated, respectively, in mLN‐iFRCs when compared to pLN‐iFRCs (Fig. 2A). To select genes most probably involved in the differential Treg‐inducing capacity of mLN‐ and pLN‐iFRCs, we decided to focus on the groups of complement proteins, cytokines and chemokines. Accordingly, the complement‐encoding genes indicated a higher expression of inflammation‐related complement molecules in pLN‐iFRCs, whereas the anti‐inflammatory *C1qtnf3* and *Fam132a* genes showed an increased expression in mLN‐iFRCs (Fig. 2B). Similarly, the pro‐inflammatory cytokine *Il6* and several chemokine‐encoding genes were expressed at higher levels in pLN‐iFRCs, while anti‐inflammatory cytokine‐encoding genes, such as *Tgfb1*, *Ltbp2* and *Pdgfc*, were significantly higher expressed in mLN‐iFRCs (Fig. 2B). In general, a considerably lower number of inflammatory mediators showed an increased expression in mLN‐iFRCs, suggesting that these cells harbor a more tolerogenic phenotype as compared to pLN‐iFRCs, which display more pro‐inflammatory properties.

### Superior Treg‐inducing capacity of mLN‐iFRCs is largely mediated by TGF‐β

**Figure 3:**
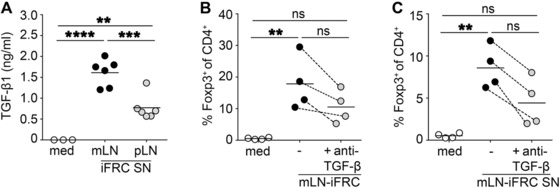
TGF‐β is a major Treg‐inducing factor of mLN‐iFRCs. (A) TGF‐β1 concentration was determined in SN of mLN‐ and pLN‐iFRCs by ELISA. Scatterplot depicts means of technical replicates pooled from two independent experiments with three independent cultures of mLN‐ and pLN‐iFRCs per experiment. Lines represent mean of depicted dots within respective groups. (B, C) CTV‐labeled naïve CD4^+^ T cells from Foxp3^hCD2^xRag2^‐/‐^xDO11.10 mice were added to mLN‐iFRCs (B) or mLN‐iFRC‐derived SNs (C) in presence of IL‐2 and anti‐CD3/CD28 Dynabeads. In part of the cultures, neutralizing antibodies directed against TGF‐β1,2,3 were added. Four days later, the frequency of de novo induced Foxp3^+^ cells among CD4^+^ T cells was determined by flow cytometry. Each dot within scatterplot depicts mean of technical replicates, and dotted lines connect corresponding mean values from the four independently performed experiments. Each line represents mean of depicted dots within respective groups. *p*‐values were calculated using one‐way ANOVA followed by Bonferroni's post‐test. ns, not significant; **p < 0.01; ***p < 0.001; ****p < 0.0001. med, medium.

Since the RNA‐seq analysis revealed an increased expression of *Tgfb1* in mLN‐iFRCs compared to pLN‐iFRCs (Fig. 2B), we next asked whether this key anti‐inflammatory cytokine, which has been reported to be instrumental for de novo Treg induction 31, 32, is responsible for the superior Treg‐inducing capacity of mLN‐iFRCs. First, we investigated whether differential expression of *Tgfb1* is translated to protein level, and measured TGF‐β1 levels in mLN‐ and pLN‐iFRC‐derived SNs. According to our expectations, TGF‐β1 levels were significantly higher in SN of mLN‐iFRCs as compared to SN collected from pLN‐iFRCs (Fig. 3A). To unravel whether the superior Treg‐inducing capacity of mLN‐iFRCs is a consequence of higher TGF‐β1 expression, neutralizing antibodies directed against TGF‐β1,2,3 were added to de novo Treg induction cultures. Exogenous TGF‐β1 was used as positive control, and its Treg‐inducing capacity could be dose‐dependently inhibited by addition of neutralizing antibodies (data not shown). When naïve CD4^+^ T cells were cultured with either mLN‐iFRCs or mLN‐iFRC‐derived SN, addition of neutralizing TGF‐β1,2,3‐specific antibodies resulted in a substantial reduction of the frequency of de novo induced Foxp3^+^ Tregs (Fig. 3B and C), albeit not reaching statistical significance. Additionally, RNA‐seq analysis revealed significantly elevated expression of *Itgb8* (integrin* *β_8_) in mLN‐iFRCs as compared to pLN‐iFRCs (log2FC ‐2.22, adjusted *p*‐value  < 0.0001, Supporting Information Fig. 2), providing a possible mechanism of processing iFRC‐derived TGF‐β1 33, 34, 35. Collectively, our data suggest that TGF‐β is a major mediator of the superior Treg‐inducing properties of mLN‐iFRCs, which furthermore display elevated expression levels of the TGF‐β‐activating integrin* *β_8_.

### mLN‐iFRC‐derived MVs are conveyors of TGF‐β

**Figure 4:**
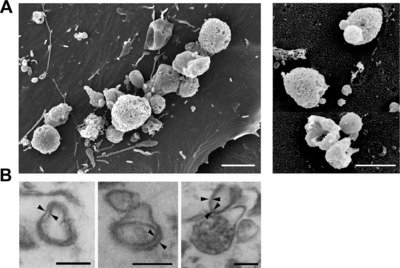
iFRCs secrete subcellular structures, corresponding to the size‐range of MVs. (A) iFRCs were cultured for 24 h, directly fixed and prepared for field emission scanning electron microscopy. mLN‐ (left, scale bar 2 μm) and pLN‐iFRC‐derived MVs (right, scale bar 1 μm) are depicted. Representative pictures are shown from three independent experiments. (B) For transmission electron microscopy analysis, MVs were isolated from SN of mLN‐iFRCs by differential centrifugation and gravity‐driven filtration. Pellets were fixed after washing and prepared for analysis. Length of scale bars corresponds to 200 nm. Black arrows indicate lipid bilayer structure of vesicles. Representative pictures are shown from the single experiment performed.

**Figure 5:**
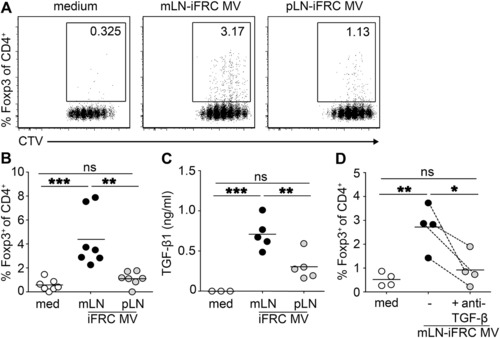
MV‐associated TGF‐β is responsible for the tolerogenic phenotype of mLN‐iFRCs. MVs were isolated from SN of mLN‐ and pLN‐iFRCs by differential centrifugation and gravity‐driven filtration. (A, B) CTV‐labeled naïve CD4^+^ T cells from Foxp3^hCD2^xRag2^‐/‐^xDO11.10 mice were cultured in presence of IL‐2, anti‐CD3/CD28 Dynabeads, and in part of the cultures MVs derived from either mLN‐ or pLN‐iFRCs were added. Four days later, the frequency of de novo induced Foxp3^+^ cells and CD4^+^ T cell proliferation was determined by flow cytometry, as shown in representative dot plots (A). Numbers in gates indicate frequencies. (B) Each dot within scatterplot depicts mean of technical replicates, and each line represents mean of depicted dots within respective groups. Data were pooled from seven independent experiments with single cultures of mLN‐ and pLN‐iFRCs per experiment; *p*‐values were calculated using one‐way ANOVA followed by Bonferroni's post‐test. ns, not significant; **p < 0.01; ***p < 0.001. (C) TGF‐β1 concentration in MVs of mLN‐ and pLN‐iFRCs was determined by ELISA. Each dot within scatterplot depicts mean of technical replicates, and lines represent mean of depicted dots within respective groups. Data were pooled from five independent experiments; *p*‐values were calculated using one‐way ANOVA followed by Bonferroni's post‐test. ns, not significant; **p < 0.01; ***p < 0.001. (D) Naïve T cells were stimulated as described above. In part of the cultures, neutralizing antibodies directed against TGF‐β1,2,3 were added. Each dot within scatterplot depicts mean of technical replicates, and dotted lines connect corresponding mean values from the four independently performed experiments. Each line represents mean of depicted dots within respective groups. *p*‐values were calculated using one‐way ANOVA followed by Bonferroni's post‐test. ns, not significant; *p < 0.05; **p < 0.01. med, medium.

As mLN‐iFRCs release increased amounts of TGF‐β1 when compared to pLN‐iFRCs, we next asked whether TGF‐β1 is associated with iFRC‐derived EVs. Using field emission scanning electron microscopy, we observed that both mLN‐ and pLN‐iFRCs can release EVs at comparable level (Fig. 4A). Similarly, we showed secretion of EVs by FRCs ex vivo isolated from mLN and pLN (Supporting Information Fig. 3A), proving that EV release is a general property of FRCs, not affected by immortalization. Presence of EVs in the pellet of iFRC‐derived SN was confirmed by transmission electron microscopy, showing an average size of 100–400 nm in diameter (Fig. 4B), corresponding to the size range of MVs 18. Next, we determined the size distribution of EVs isolated from mLN‐ and pLN‐iFRC‐derived SN by tunable resistive pulse sensing (TRPS) analysis, and confirmed that both are in the size range of MVs with a diameter of 100–1000 nm (Supporting Information Fig. 3B). Flow cytometric analysis revealed substantial expression of EV markers such as annexin* *V and CD63 on both mLN‐ and pLN‐iFRC‐derived MVs, whereas expression of CD9 and gp38 was rather low (Supporting Information Fig. 3C and data not shown). CD40, which fine‐tunes CD4^+^ **T* *cell responses 36 and is often found on MVs 37, showed similar expression levels on mLN‐ and pLN‐iFRC‐derived MVs (Supporting Information Fig. 3C). After confirming that mLN‐ and pLN iFRC‐derived vesicle samples truly contain MVs, we added them to de novo Treg induction cultures. A significantly increased frequency of de novo induced Foxp3^+^ Tregs was observed in cultures where mLN‐iFRC‐derived MVs were added when compared to cultures receiving pLN‐iFRC‐derived MVs (Fig. 5A and B), suggesting that iFRC‐released MVs are involved in intercellular communication between LN stromal cells and CD4^+^ T cells.

To assess the amount of TGF‐β associated with mLN‐ and pLN‐iFRC‐derived MVs, the concentration in isolated MV preparations was determined. MVs isolated from mLN‐iFRCs carried significantly higher TGF‐β1 levels as compared to MVs of pLN‐iFRCs (Fig. 5C), however the assay did not allow discriminating between intravesicular or surface‐bound TGF‐β. Finally, we tested the functional importance of high TGF‐β content in mLN‐iFRC‐derived MV preparations. Addition of neutralizing antibodies directed against TGF‐β1,2,3 to naïve CD4^+^ T cells cultured in presence of mLN‐iFRC‐derived MVs resulted in a significantly reduced frequency of de novo induced Foxp3^+^ Tregs (Fig. 5D). Taken together, our data reveal that TGF‐β is associated with MVs released by mLN‐iFRCs and promotes the superior Treg‐inducing capacity of mLN‐iFRCs.

## Discussion

Maintenance of intestinal homeostasis requires multiple layers of regulatory mechanisms. Accumulating evidence suggests a major contribution of Foxp3^+^ Tregs to mucosal tolerance 38, and recent publications reported a particularly efficient de novo Foxp3^+^ Treg induction in gut‐draining as compared to skin‐draining LNs 10, 12, 39. Emerging evidence indicates that the stromal compartment of gut‐draining LNs, with FRCs dominating in the T‐cell zone 40, accounts for the tolerogenic capacity of these LNs in a DC‐dependent and also ‐independent manner via direct interaction with T cells 12. However, the exact mechanism of how FRCs modulate Treg induction is unknown. Here, we provide evidence that FRCs can directly influence T cell differentiation and that mLN‐iFRCs have a superior Treg‐inducing capacity when compared to pLN‐iFRCs, mediated by cell‐derived MVs associated with TGF‐β. For the first time, this finding demonstrates an intercellular crosstalk between FRCs and T cells via MVs and reveals a novel mechanism of immune regulation taking place in gut‐draining LNs. However, it has to be mentioned that most experiments were performed with immortalized cells, making it necessary to confirm in vivo relevance using ex vivo isolated FRCs in future studies.

Several studies indicate a role of FRCs in immune tolerance, including FRC‐constrained expression of peripheral tissue antigens 41, 42, 43 and acquisition of peptide‐MHCII complexes from DCs via exosomes 44, finally resulting in an amelioration of effector T‐cell responses. In addition, FRCs can limit T‐cell responses via release of nitric oxide, produced by the inducible nitric oxide synthase enzyme, whose expression is increased under inflammatory conditions 45, 46. Additionally, IFN‐γ can also stimulate programmed death ligand‐1 and indoleamine 2,3‐dioxygenase expression of FRCs 45, 46, both affecting peripheral Treg induction. Furthermore, high expression levels of RALDH in mLN‐FRCs 14, 17, 47 and CD103^+^ DCs is responsible for shaping the tolerogenic microenvironment of gut‐draining mLNs.

To decipher molecular details of mucosal tolerance in context of LN stromal cells and better understand the dialogue between FRCs and Tregs, we generated immortalized stromal cell lines with differential Treg‐inducing capacity and analyzed expression of their secreted molecules. In line with the previously described imprinted tolerogenic phenotype of gut‐draining mLNs 12, several pro‐inflammatory mediators were downregulated in mLN‐iFRCs when compared to pLN‐iFRCs. Among the upregulated secreted molecules of mLN‐iFRCs, the cytokine‐like C‐type lectin *Clec11a* might contribute to the modulation of immune responses. This lectin functions as a growth factor, and acts synergistically with IL‐3, GM‐CSF and Flt3 ligand in driving DC development 48, suggesting a potential interaction between mLN‐FRCs and migratory DCs via Clec11a, finally shaping tolerogenic DC functions and establishing mucosal tolerance. Moreover, the C1q/TNF‐related protein family member *C1qtnf3* and the adipolin *Fam132a*, both upregulated in mLN‐iFRCs compared to pLN‐iFRCs, have been reported to confer anti‐inflammatory activities 49, 50, 51, and thus further support the tolerogenic phenotype of mLN‐iFRCs. Upregulated expression of *Pdgfc* in mLN‐iFRCs could also be related to tolerogenic mechanisms, as *Pdgfc* has been recently described to favor Treg induction indirectly via DC modulation 52.

Interestingly, RNA‐seq analysis of the stromal cell lines also revealed an upregulation of *Tgfb1* in mLN‐iFRCs when compared to pLN‐iFRCs. This observation fits to the previously described microarray data analyzing ex vivo isolated FRCs, where *Tgfb1* expression was upregulated in mLN‐FRCs compared to pLN‐FRCs 47. On the other hand, previous results showed that exogenous TGF‐β does not affect the in vitro Treg‐inducing capacity of ex vivo stromal* *cells 12. However, these in vitro cultures of unsorted LN stromal* *cells most probably contained a mixture of FRCs, lymphatic endothelial cells, blood endothelial cells, and double negative stromal* *cells. This could easily blur gene expression in FRCs, and consequently did not provide proper information about the direct mechanism of FRC‐driven Treg induction. Since TGF‐β plays a pivotal role in peripheral generation and expansion of Foxp3^+^ Tregs 32, 53, we aimed to clarify whether release of TGF‐β1 is responsible for the tolerogenic phenotype of mLN‐FRCs. In line with results of the RNA‐seq analysis, TGF‐β1 concentration in SN of mLN‐iFRCs was significantly higher as compared to SN of pLN‐iFRCs. Moreover, the superior Treg‐inducing capacity of both mLN‐iFRCs and mLN‐iFRC‐derived SNs was significantly reduced by the highest concentration of TGF‐β neutralizing antibodies. Therefore, these findings suggest that the capacity of mLN‐FRCs to secrete high amounts of the anti‐inflammatory cytokine TGF‐β is critically contributing to the stromal cell‐driven efficient Treg induction.

Recent advances revealed the molecular composition of EVs, and their ability to horizontally transfer biologically active molecules 18, 26, 28. Therefore, we assumed that TGF‐β is not simply cytokine secreted by iFRCs, but this anti‐inflammatory cytokine is associated with stromal cell‐derived EVs. To date, several vesicle‐associated cytokines, such as IL‐1α, IL‐1β, IL‐6, IL‐18, IL‐32 and TNF‐α 25 have been described. TGF‐β is also associated with thymocyte‐ and tumor‐derived EVs and involved in Treg induction 24, 54, 55. Furthermore, DC‐derived exosomes were shown to transfer MHCII molecules to LN‐resident stromal cells, thereby inducing CD4^+^ T cell‐mediated tolerance 44, 46, and apoptotic neutrophil‐derived EVs were recently reported to possess suppressive capacity on human T cells 56. Thus, we raised the question whether FRCs can release EVs and whether mLN stromal cell‐induced tolerance can be attributed to these subcellular structures. Accordingly, we here showed for the first time that both mLN‐ and pLN‐iFRCs release MVs, similar to ex vivo FRCs. Moreover, mLN‐iFRC‐derived MVs have a profound Treg‐inducing capacity as compared to vesicles isolated from pLN‐iFRCs due to vesicle‐associated TGF‐β.

Since TGF‐β is produced as an inactive complex, it is critical to understand how FRC‐derived TGF‐β is activated. We found an elevated expression of *Itgb8* in mLN‐iFRCs as compared to pLN‐iFRCs, suggesting that similar to intestinal DCs and Tregs 33, 34, 35 iFRCs can activate TGF‐β in an integrin* *β_8_‐dependent manner. This observation is in line to previous findings from ex vivo mLN and pLN stromal* *cells 12. In addition, MVs are known to carry molecules of donor origin 18, making it likely that mLN‐iFRC‐derived MVs carry integrin β_8_ and contribute to TGF‐β activation when CD4^+^ naïve T cells were cultured with iFRC‐derived MVs only. Finally, it is also conceivable that de novo induced Tregs represent a second line of TGF‐β activation due to their expression of *Itgb8* 34.

Taken together, iFRCs originating from gut‐draining mLNs harbor a superior Treg‐inducing ability as compared to iFRCs derived from skin‐draining pLNs. These cell lines provide a unique tool to investigate intercellular communication networks between FRCs and immune cell compartments. Moreover, our study provided first insights into molecular details of the direct dialogue between FRCs and differentiating T cells. We emphasized the importance of FRC‐derived TGF‐β for de novo Treg induction, and its horizontal transfer via iFRC‐derived vesicles. Efforts to understand novel ways of intercellular communication in addition to the known pathways including cell‐cell contact or soluble mediators could provide avenues for future development of novel vaccination approaches and therapies to treat autoimmune disorders.

## Materials and methods

### Mouse strains

Foxp3^hCD2^xRag2^‐/‐^xDO11.10 (BALB/c) and wild‐type BALB/c mice were bred and housed under specific pathogen‐free conditions at Helmholtz Centre for Infection Research (Braunschweig, Germany). Gender‐ and age‐matched mice were used in all experiments. Mice were housed and handled with appropriate care and welfare in accordance with good animal practice as defined by FELASA and national animal welfare body GV‐SOLAS under supervision of the institutional animal welfare officer. All efforts were made to minimize suffering.

### Flow cytometry

Fluorochrome‐conjugated anti‐hCD2 (TS1/8), anti‐CD4 (RM4‐5), anti‐CD9 (MZ3), anti‐CD24 (M1/69), anti‐CD25 (7D4), anti‐CD31 (390), anti‐CD40 (1C10), anti‐CD45 (30‐F11), anti‐CD45.2 (104), anti‐CD63 (NVG‐2) and anti‐gp38 (8.1.1) antibodies were purchased from eBioscience, BioLegend and BD. Dead cells were excluded utilizing LIVE/DEAD Fixable Blue Dead Cell Stain (Thermo Fisher Scientific) and scatter properties. Cells were analyzed on LSR Fortessa with Diva software v8.0.1 (BD), and data analysis was performed with FlowJo software v9.9.3 (TreeStar).

### Immortalized stromal cell lines

FRCs were isolated ex vivo from pLNs and mLNs of BALB/c mice as described previously 57. In brief, LNs were digested in RPMI 1640‐medium (Thermo Fisher Scientific) supplemented with 0.2 mg/ml collagenase P, 0.15 U/mL dispase and 0.2 mg/ml DNase I (all purchased from Roche) and subsequently kept at 4°C in PBS containing 0.2% BSA and 5 mM EDTA (Sigma‐Aldrich). Hematopoietic cells were stained with anti‐CD45‐APC antibody, followed by labeling with anti‐APC MicroBeads and enrichment of CD45^‐^ cells using the autoMACS separation system (Miltenyi Biotec). Subsequently, CD45^‐^CD24^‐^CD31^‐^gp38^+^ FRCs were sorted on FACSAriaII (100 μm nozzle, purity > 91–97%). 2 × 10^4^ FRCs were plated on 24‐well plates (Thermo Fisher Scientific) coated with 0.05 mg/mL fibronectin (Roche). Immortalization was performed via lentiviral transduction of a Tet‐inducible SV40 T‐antigen as described previously 30, 58. After 48 h transfection, cells were selected for resistance against 0.4 mg/ml G418 (Thermo Fisher Scientific). For cell expansion, proliferation of mLN‐ and pLN‐iFRCs was induced by addition of 2 μg/mL doxycyclin (AppliChem), and proliferation of iFRCs was stopped in all experimental conditions via doxycyclin removal. Three independently generated pairs of mLN‐ and pLN‐iFRC cell lines were tested for Treg induction, and any pLN‐iFRC line harbored a remarkable Treg‐inducing capacity. Among mLN‐iFRCs, the cell line most closely resembling the differential Treg inducing‐capacity of ex vivo stromal cells 12, was chosen for all further analyses and paired with one pLN‐iFRC line.

### SN harvesting and MV isolation from iFRCs

iFRC‐derived SNs and MVs were isolated based on previously described protocols 18, 26. Briefly, mLN‐ and pLN‐iFRCs were cultured for 48 hours in a 6‐well plate (3.5 × 10^5^ cells per well in 1.5 mL chemically defined serum‐free hematopoietic cell medium X‐VIVO, Lonza). Conditioned medium was collected, and remaining cells or cell debris were removed via two sequential centrifugation steps (450 × *g*, 8 min) followed by filtration via 5 μm filters (Merck Millipore). Part of this filtered medium was kept as SN and either applied immediately for Treg induction assays or stored at −80°C for TGF‐β1 ELISA. From the remaining part, apoptotic bodies were pelleted by centrifugation (2000 × *g*, 20 min) followed by filtration via 0.8 μm filters (Sigma‐Aldrich). From the apoptotic body‐free SN, MVs were pelleted by centrifugation (20 500 × *g*, 60 min), and washed to remove excessive soluble factors of the SN. For Treg induction assays, MVs were collected in X‐VIVO and applied immediately. In general, MVs isolated from pooled SN of a complete 6‐well plate were resuspended in 600–900 μL X‐VIVO. For TGF‐β1 ELISA, MVs were resuspended in sterile distilled water and stored at −80°C.

### In vitro Treg induction

For de novo Treg induction in presence of iFRCs, 3 × 10^4^ mLN‐ or pLN‐iFRCs were plated into each well of a 96‐well plate in 150 μL X‐VIVO and cultured overnight. Naïve CD4^+^ T cells were enriched from secondary lymphoid organs of Foxp3^hCD2^xRag2^‐/‐^xDO11.10 mice using CD4 (L3T4) MicroBeads and autoMACS separation. 10^5^ naïve CD4^+^ T cells labeled with proliferation dye Cell Trace Violet (CTV, ThermoFisher Scientific) were added to iFRCs in 50 μL X‐VIVO containing 10 ng/mL IL‐2 and 0.5 × 10^5^ Dynabeads mouse T activator CD3/CD28 (Thermo Fisher Scientific). Cells were co‐cultured for 4 days, and frequency of Foxp3^+^ cells and degree of T‐cell proliferation was determined by flow cytometry. For de novo Treg induction in presence of iFRC‐derived SNs or MVs, SNs and MVs were always freshly generated. 150 μL SN or 150 μL MVs were added to 10^5^ naïve CD4^+^ T cells isolated and labeled as described above. For blocking experiments, indicated concentrations of neutralizing antibodies directed against TGF‐β1,2,3 (1D11) (R&D Systems) or isotype antibodies (IgG1) (BioXCell) were added. Cells were stimulated, cultured and analyzed as described above.

### RNA‐seq analysis

Total RNA was isolated from mLN‐ and pLN‐iFRCs using the AllPrep DNA/RNA kit (Qiagen). Agilent Technologies 2100 Bioanalyzer was used to confirm quality and integrity of total RNA. Poly A‐containing mRNA was purified via poly T oligo‐attached magnetic beads (Illumina). The mRNA was subsequently subjected to library preparation using Script Seq v2 Library preparation kit (Illumina). Deep sequencing was performed on Illumina HiSeq2500 using 50 bp single reads. The FastQC tool was used to assess sequenced libraries for read quality, which were then aligned versus the mouse reference genome (assembly: GRCm38) by using splice junction mapper Tophat2 v1.2.0 59 with default parameterization. The htseq‐count program was utilized to quantify reads aligned to annotated genes 60. Determined read counts served as input to DESeq2 61 for pairwise detection and quantification of differential gene expression. Only genes with annotated official *Gene Symbol* were included into heatmaps. From the raw read counts RPKM (reads per kilobase maximal transcript length per million mapped reads) values were computed for each library. The Gene Expression Omnibus (GEO) accession number for RNA‐seq data reported in this paper is GSE93670.

### TGF‐β ELISA

TGF‐β1 concentration in SN and MV samples of mLN‐ and pLN‐iFRCs was determined by the Mouse TGF‐beta 1 Quantikine ELISA Kit (R&D) according to manufacturer`s instructions. Both SN and MV samples were stored at −80°C prior use and 50 μL per sample were applied. Absorbance was measured at 450/540 nm on Infinite 200 microtiter plate reader (Tecan).

### Field emission scanning electron microscopy (FESEM)

To characterize vesicles released by iFRCs or ex vivo isolated FRCs, cells were cultured in X‐VIVO for 24 h and directly fixed in cultures plates with 5% formaldehyde and 2% glutaraldehyde in HEPES buffer (0.1 M HEPES, 10 mM CaCl_2_, 10 mM MgCl_2_ and 0.09 M sucrose, pH 6.9) overnight at 7°C, washed with TE buffer (20 mM TRIS, 2 mM EDTA, pH 6.9), and then dehydrated in a graded series of acetone (10, 30, 50, 70, 90, 100%; 10 min on ice each step). Samples in the 100% acetone step were allowed to reach room temperature before another change in 100% acetone. Samples were then subjected to critical‐point drying with liquid CO_2_ (CPD 300, Leica). Dried samples were covered with a gold‐palladium film by sputter coating (SCD 500 Bal‐Tec) before examination in a field emission scanning electron microscope Zeiss Merlin using the Everhart‐Thornley SE‐detector and the Inlens SE‐detector in a 25:75 ratio with an acceleration voltage of 5 kV. Contrast and brightness were adjusted with Adobe Photoshop CS5.

### Ultrathin sections and transmission electron microscopy

To confirm the presence of vesicular structures in the iFRC MV samples, MVs were isolated as described above followed by an additional washing step in 100 mM HEPES buffer. Supernatants were carefully removed and pellets were fixed with 1% glutaraldehyde in HEPES buffer, washed twice with buffer and treated with1% aqueous osmium in 0.08 M cacodylate buffer for 1.5 h at room temperature. Samples were immobilized in 1.5% agar and dehydrated with a graded series of acetone (10, 30, 50, 70, 90 and 100%; 30 min each step). Samples were left overnight in 2% uranyl acetate in the 70% acetone step at 7°C. Samples were then infiltrated with an epoxy resin (Agar Low Viscosity Resin, hard formula, Agar Scientific). Ultrathin sections were cut with a diamondknife, picked up with butvar‐coated 300 mesh grids, and counterstained with 4% aqueous uranyl acetate. Samples were examined in a TEM910 transmission electron microscope (Carl‐Zeiss) at an acceleration voltage of 80 kV. Images were taken at calibrated magnifications using a line replica. Images were recorded digitally with a Slow‐Scan CCD‐Camera (1024 × 1024, ProScan) with ITEM‐Software (Olympus Soft Imaging Solutions). Contrast and brightness were adjusted with Adobe Photoshop CS5.

### Tunable resistive pulse sensing (TRPS)

MVs were isolated from mLN‐ and pLN‐iFRCs as described above, followed by TRPS analysis using qNano instrument (IZON Sciences Ltd.) as described previously 62. Carboxylated polystyrene particle standards were used to determine the vesicle size. Main characteristics of calibration particles and TRPS measurement are summarized in Supporting Information Table 2. Dilution of samples varied between 10 and 40 times. We counted at least 500 events/sample. Izon Control Suite software (v3.1, Izon Science) enabled to convert number and magnitude of measured pore blockades to particle diameter and concentration.

### Flow cytometric analysis of iFRC‐derived MVs

MV samples isolated from mLN‐ and pLN‐iFRCs were isolated as described above and resuspended in PBS, followed by conjugation to aldehyde/sulfate latex beads (Life Technologies), blocking with 100 mmol/L glycine and 1 w/v % bovine serum albumin (BSA) in PBS at room temperature. After centrifugation (1000 × *g*, 10 min), the pellet was resuspended in PBS or annexin V binding buffer (10 mM HEPES/NaOH, 140 mM NaCl, 2.5 mM CaCl_2_ pH 7.4) and incubated with annexin V‐FITC (BioLegend) or antibodies at room temperature for 30 min, followed by an additional washing step at 1000 × *g* for 10 min. Threshold of negative staining was obtained with BSA‐coated beads incubated with annexin V‐FITC or respective antibodies.

### Statistical analysis

Group sizes were estimated according to a presumed standard deviation (SD) and an expected type I error of <0.05. The sample size was adjusted, if required, based on initial results. The comparison of more than two groups was performed by one‐way ANOVA followed by Bonferroni's post‐test. All data are presented as mean or mean ± SD, and *p*‐values < 0.05 are considered as significant (*p < 0.05; **p < 0.01; ***p < 0.001; ****p < 0.0001). Prism software (GraphPad) was applied for statistical analyses and heatmaps were generated with the R package pheatmap.

## Conflict of interest

The authors declare no commercial or financial conflict of interest.

**Abbreviations**
DC: dendritic cells
EV: extracellular vesicles
FRC: fibroblastic reticular cells
iFRC: immortalized fibroblastic reticular cells
mLN: mesenteric lymph nodes
MV: microvesicles
pLN: peripheral lymph nodes
Treg: regulatory T cells
SN: supernatant

## Supporting information

Peer review correspondence

Supplementary Table 1

**Supplementary Table 2**. Main characteristics of the tunable resistive pulse sensing (TRPS) measurement and calibration particles. MVs were isolated from mLN‐ and pLN‐iFRCs as described before, followed by TRPS analysis using qNano instrument (IZON Sciences Ltd.). Characteristics of the carboxylated polystyrene particles (all from IZON) are summarized. During the measurements, the stretch of the TRPS nanopore and the applied voltage remained the same, thus keeping the measurements within the optimal range.

**Supporting Information Fig. 1**. Gating strategy used to determine Treg‐inducing capacity of mLN‐ or pLN‐iFRCs. CTV‐labeled naïve CD4+ T cells from Foxp3hCD2xRag2‐/‐xDO11.10 mice were in vitro cultured with iFRCs in the presence of IL‐2 and anti‐CD3/CD28 Dynabeads. Four days later, cells were analyzed by flow cytometry. After gating on lymphocytes (left, upper row), and excluding douplets (middle and right, upper row), living and proliferating CD4+ T cells (left and middle of lower row, respectively) were further analyzed. The frequency of de novo induced Foxp3+ cells among proliferating CD4+ T cells was determined (right, lower row), as shown in representative dot plots. Numbers in gates indicate frequencies. The same gating strategy was used for all Treg‐induction assays throughout the study. CTV, Cell Trace Violet; LD, LIVE/DEAD Fixable Blue Dead Cell Stain.
**Supporting Information Fig. 2**. Differential expression of *Itgb8* in mLN‐ and pLN‐iFRCs. RNA‐seq analysis was performed on mLN‐ and pLN‐iFRCs. Genes with |log2 (FC)| ≥ 1 and q value ≤ 0.05 were considered differentially expressed. Heatmap represents the differential expression of *Itgb8* in mLN‐ and pLN‐iFRCs. Color coding is based on RPKM normalized count values. Data from three independent cultures of mLN‐ and pLN‐iFRCs are depicted. FC, fold change; RPKM, reads per kilobase maximal transcript length per million mapped reads.
**Supporting Information Fig. 3**. Characterization of mLN‐ and pLN‐iFRC‐derived MVs. (A) FRCs were isolated ex vivo from pLN and mLN of BALB/c mice by enzymatic digestion and directly FACS sorted onto fibronectin‐coated chamber slides. After culturing for 24 hours, FRCs were directly fixed and prepared for field emission scanning electron microscopy. Ex vivo mLN‐ (left) and pLN‐ (right) FRC‐derived MVs are depicted. Scale bars correspond to 2 μm. (B, C) MVs were isolated from 24h SN of mLNand pLN‐iFRCs via differential centrifugation and gravity‐driven filtration. (B) The size distribution of mLN‐ and pLN‐iFRC MVs was determined by tunable resistive pulse sensing analysis. Representative graph is shown from the measurement with the NP400 nanopore membrane of a single experiment. (C) After coupling mLN‐ (upper row) and pLN‐ (lower row) iFRC MVs to aldehyde/sulphate latex beads and blocking the remaining binding capacity with BSA, beads were incubated with antibodies against EV‐specific markers and analyzed by flow cytometry. Numbers indicate geometric mean of labeled MV‐coated beads (black) compared to BSA‐coated control beads incubated with the respective antibodies (grey).
