# Supplementary material for: Mesenteric lymph node stromal cell‐derived extracellular vesicles contribute to peripheral de novo induction of Foxp3+ regulatory T cells
Source: Eur J Immunol. 2017 Sep 15;47(12):2142–52. doi: 10.1002/eji.201746960 (PMC5724490; doi:10.1002/eji.201746960)
Supplement: Supplementary file 3 — Supplementary Table 2. Main characteristics of the tunable resistive pulse sensing (TRPS) measurement and calibration particles. MVs were isolated from mLN‐ and pLN‐iFRCs as described before, followed by TRPS analysis using qNano instrument (IZON Sciences Ltd.). Characteristics of the carboxylated polystyrene particles (all from IZON) are summarized. During the measurements, the stretch of the TRPS nanopore and the applied voltage remained the same, thus keeping the measurements within the optimal range. [file EJI-47-2142-s003.docx]

| Nanopore type | Calibration bead | Mean Diameter (nm) | Dilution factor | Particle Count | relative blockade magnitude (dI/I) | Average Current (nA) | Applied Stretch (mm) | Applied Voltage (V) | Pressure |
| --- | --- | --- | --- | --- | --- | --- | --- | --- | --- |
|  |  |  |  |  |  |  |  |  |  |
| NP200 | CPC100B (before measurement) | 110 | 1000 | 1078 | 0.10-0.13 | 87.81 | 45.71 | 0.34 | 10 |
|  | CPC200B (before measurement) | 210 | 1000 | 2113 | 0.18-0.25 | 97.62 |  |  |  |
|  | CPC200B (after measurement) | 210 | 1000 | 2103 | 0.18-0.25 | 84.08 |  |  |  |
|  | CPC400G (before measurement) | 340 | 750 | 1993 | 0.80-1.10 | 102.84 |  |  |  |
|  | CPC400G (after measurement) | 340 | 1000 | 2315 | 0.80-1.10 | 91.91 |  |  |  |
| NP400 | CPC400G (before measurement) | 340 | 2000 | 1057 | 0.10-0.25 | 119.94 | 45.73 | 0.30 | 10 |
|  | CPC400G (after measurement) | 340 | 4000 | 1581 | 0.10-0.25 | 102.14 |  |  |  |
|  | CPC800E (before measurement) | 710 | 1000 | 675 | 1.00-2.00 | 112.01 |  |  |  |

**Supplementary Table 2.** Main characteristics of the tunable resistive pulse sensing (TRPS) measurement and calibration particles. MVs were isolated from mLN- and pLN‑iFRCs as described before, followed by TRPS analysis using qNano instrument (IZON Sciences Ltd.). Characteristics of the carboxylated polystyrene particles (all from IZON) are summarized. During the measurements, the stretch of the TRPS nanopore and the applied voltage remained the same, thus keeping the measurements within the optimal range.
