# Supplementary material for: Mesenteric lymph node stromal cell‐derived extracellular vesicles contribute to peripheral de novo induction of Foxp3+ regulatory T cells
Source: Eur J Immunol. 2017 Sep 15;47(12):2142–52. doi: 10.1002/eji.201746960 (PMC5724490; doi:10.1002/eji.201746960)
Supplement: Supplementary file 4 — Supporting Information Fig. 1. Gating strategy used to determine Treg‐inducing capacity of mLN‐ or pLN‐iFRCs. CTV‐labeled naïve CD4+ T cells from Foxp3hCD2xRag2‐/‐xDO11.10 mice were in vitro cultured with iFRCs in the presence of IL‐2 and anti‐CD3/CD28 Dynabeads. Four days later, cells were analyzed by flow cytometry. After gating on lymphocytes (left, upper row), and excluding douplets (middle and right, upper row), living and proliferating CD4+ T cells (left and middle of lower row, respectively) were further analyzed. The frequency of de novo induced Foxp3+ cells among proliferating CD4+ T cells was determined (right, lower row), as shown in representative dot plots. Numbers in gates indicate frequencies. The same gating strategy was used for all Treg‐induction assays throughout the study. CTV, Cell Trace Violet; LD, LIVE/DEAD Fixable Blue Dead Cell Stain. Supporting Information Fig. 2. Differential expression of Itgb8 in mLN‐ and pLN‐iFRCs. RNA‐seq analysis was performed on mLN‐ and pLN‐iFRCs. Genes with |log2 (FC)| ≥ 1 and q value ≤ 0.05 were considered differentially expressed. Heatmap represents the differential expression of Itgb8 in mLN‐ and pLN‐iFRCs. Color coding is based on RPKM normalized count values. Data from three independent cultures of mLN‐ and pLN‐iFRCs are depicted. FC, fold change; RPKM, reads per kilobase maximal transcript length per million mapped reads. Supporting Information Fig. 3. Characterization of mLN‐ and pLN‐iFRC‐derived MVs. (A) FRCs were isolated ex vivo from pLN and mLN of BALB/c mice by enzymatic digestion and directly FACS sorted onto fibronectin‐coated chamber slides. After culturing for 24 hours, FRCs were directly fixed and prepared for field emission scanning electron microscopy. Ex vivo mLN‐ (left) and pLN‐ (right) FRC‐derived MVs are depicted. Scale bars correspond to 2 μm. (B, C) MVs were isolated from 24h SN of mLNand pLN‐iFRCs via differential centrifugation and gravity‐driven filtration. (B) The size distribution of m [file EJI-47-2142-s004.pdf]

European Journal of Immunology

Supporting Information
for
DOI 10.1002/eji.201746960

Maria Pasztoi, Joern Pezoldt, Michael Beckstette, Christoph Lipps, Dagmar Wirth, Manfred Rohde, Krisztina Paloczi, Edit Iren Buzas and Jochen Huehn

Mesenteric lymph node stromal cell-derived extracellular vesicles contribute to peripheral de novo induction of Foxp3^{+} regulatory T cells

© 2017 The Authors. *European Journal of Immunology* Published by WILEY-VCH Verlag GmbH & Co. KGaA, Weinheim.

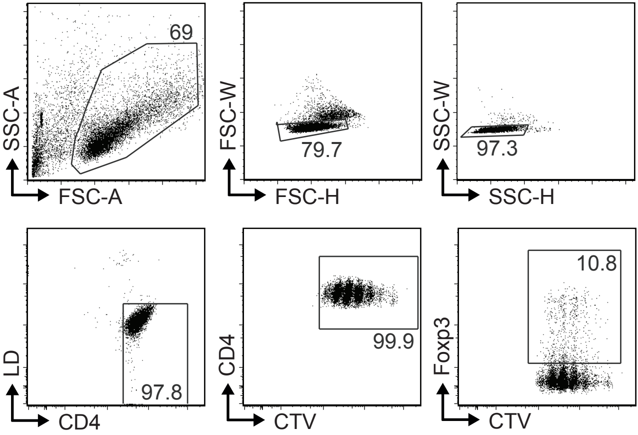

Supplementary Figure 1. Gating strategy used to determine Treg-inducing capacity of mLN- or pLN-iFRCs. CTV-labeled naïve $CD4^+$ T cells from $Foxp3^{hCD2}$x$Rag2^{-/-}$xDO11.10 mice were *in vitro* cultured with iFRCs in the presence of IL-2 and anti-CD3/CD28 Dynabeads. Four days later, cells were analyzed by flow cytometry. After gating on lymphocytes (left, upper row), and excluding douplets (middle and right, upper row), living and proliferating $CD4^+$ T cells (left and middle of lower row, respectively) were further analyzed. The frequency of *de novo* induced $Foxp3^+$ cells among proliferating $CD4^+$ T cells was determined (right, lower row), as shown in representative dot plots. Numbers in gates indicate frequencies. The same gating strategy was used for all Treg-induction assays throughout the study. CTV, Cell Trace Violet; LD, LIVE/DEAD Fixable Blue Dead Cell Stain.

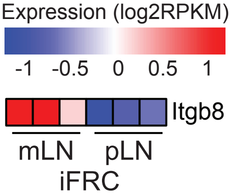

Supplementary Figure 2. Differential expression of *Itgb8* in mLN- and pLN-iFRCs. RNA-seq analysis was performed on mLN- and pLN-iFRCs. Genes with |log2 (FC)| ≥ 1 and q value ≤ 0.05 were considered differentially expressed. Heatmap represents the differential expression of *Itgb8* in mLN- and pLN-iFRCs. Color coding is based on RPKM normalized count values. Data from three independent cultures of mLN- and pLN-iFRCs are depicted. FC, fold change; RPKM, reads per kilobase maximal transcript length per million mapped reads.

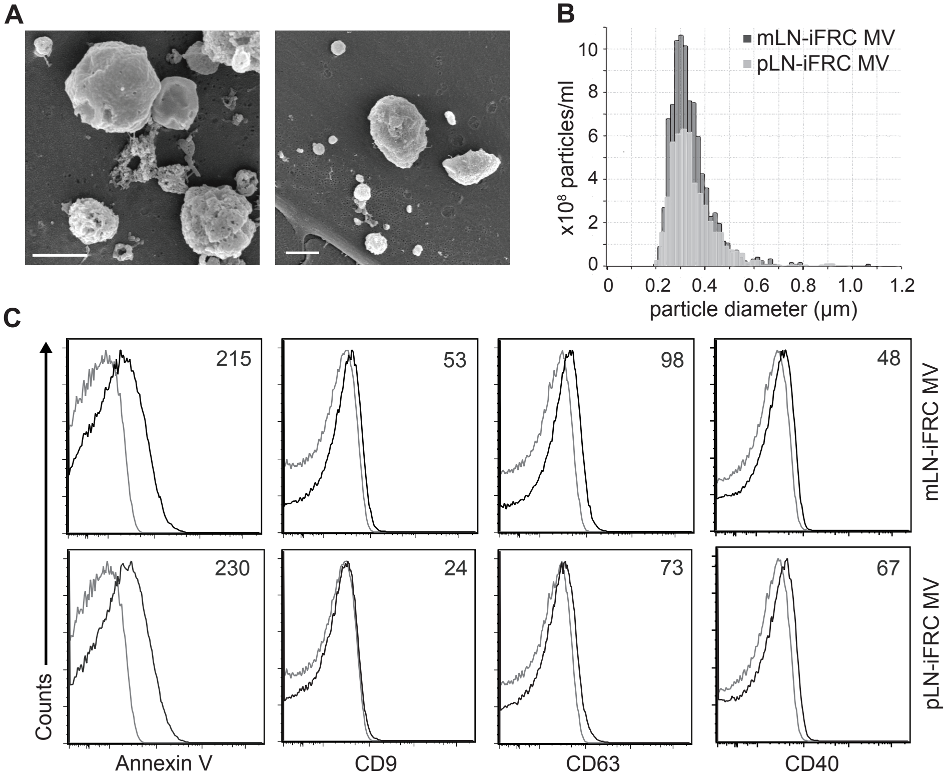

Supplementary Figure 3. Characterization of mLN- and pLN-iFRC-derived MVs. (A) FRCs were isolated *ex vivo* from pLN and mLN of BALB/c mice by enzymatic digestion and directly FACS sorted onto fibronectin-coated chamber slides. After culturing for 24 hours, FRCs were directly fixed and prepared for field emission scanning electron microscopy. *Ex vivo* mLN- (left) and pLN- (right) FRC-derived MVs are depicted. Scale bars correspond to 2 µm. (B, C) MVs were isolated from 24h SN of mLN- and pLN-iFRCs via differential centrifugation and gravity-driven filtration. (B) The size distribution of mLN- and pLN-iFRC MVs was determined by tunable resistive pulse sensing analysis. Representative graph is shown from the measurement with the NP400 nanopore membrane of a single experiment. (C) After coupling mLN- (upper row) and pLN- (lower row) iFRC MVs to aldehyde/sulphate latex beads and blocking the remaining binding capacity with BSA, beads were incubated with antibodies against EV-specific markers and analyzed by flow cytometry. Numbers indicate geometric mean of labeled MV-coated beads (black) compared to BSA-coated control beads incubated with the respective antibodies (grey).
