# Supplementary material for: Mesenteric lymph node stromal cell‐derived extracellular vesicles contribute to peripheral de novo induction of Foxp3+ regulatory T cells
Source: Eur J Immunol. 2017 Sep 15;47(12):2142–52. doi: 10.1002/eji.201746960 (PMC5724490; doi:10.1002/eji.201746960)
Supplement: Supplementary file 1 — Peer review correspondence [file EJI-47-2142-s001.pdf]

Mesenteric lymph node stromal cell derived extracellular vesicles contribute to peripheral de novo induction of Foxp3+ regulatory T cells

Maria Pasztoi, Joern Pezoldt, Michael Beckstette, Christoph Lipps, Dagmar Wirth, Manfred Rohde, Krisztina Paloczi, Edit Iren Buzas and Jochen Huehn

Correspondence: Prof. Jochen Huehn, Experimental Immunology, Helmholtz Centre for Infection Research, 38124 Braunschweig, Germany.

Review Timeline:	Submission date:	23-Jan-2017
	First Editorial decision:	17-Mar-2017
	First Revision received:	12-Jun-2017
	Second Editorial decision:	30-Jun-2017
	Final Revision received:	12-Jul-2017
	Accepted:	16-Aug-2017

Handling Executive Committee member: Prof. Shimon Sakaguchi

Please note that the correspondence below does not include the standard editorial instructions regarding preparation and submission of revised manuscripts, only the scientific revisions requested and addressed.

First Editorial Decision

17-Mar-2017

Dear Prof. Huehn,

We are sorry for a delay in the peer review of you Manuscript ID eji.201746960 entitled " Mesenteric lymph node stromal cell derived extracellular vesicles contribute to peripheral de novo induction of Foxp3+ regulatory T cells" which you submitted to the European Journal of Immunology has been reviewed. Two of our referees had different opinion and we had to seek for an opinion of a third referee. The comments of the referees are included at the bottom of this letter.

A revised version of your manuscript that takes into account the comments of the referees will be

reconsidered for publication. Should you disagree with any of the referees' concerns, you should address this in your point-by-point response and provide solid scientific reasons for why you will not make the requested changes.

You should also pay close attention to the editorial comments included below. **In particular, please edit your figure legends to follow Journal standards as outlined in the editorial comments. Failure to do this will result in delays in the re-review process.**

Please note that submitting a revision of your manuscript does not guarantee eventual acceptance, and that your revision will be re-reviewed by the referee(s) before a decision is rendered.

If the revision of the paper is expected to take more than three months, please inform the editorial office. Revisions taking longer than six months may be assessed by new referees to ensure the relevance and timeliness of the data.

Once again, thank you for submitting your manuscript to European Journal of Immunology and we look forward to receiving your revision.

Yours sincerely,
Nadja Bakocevic

On behalf of
Prof. Shimon Sakaguchi

Dr. Nadja Bakocevic
Editorial Office
European Journal of Immunology
e-mail: ejied@wiley.com
www.eji-journal.eu

****

Reviewer: 1

Comments to the Author
1. This paper only describes the genetic and functional properties of a limited number of cells lines generated in vitro from fibroblastic reticular cells from mesenteric LN versus skin draining LN. It is unclear

if the properties of these cells lines actualy are similar to the properties of FRCs in vivo or freshly explanted in vitro. For example, the authors demonstrate that the lines produce extracellular vesicles containing TGF-b. Is this a property of transformed FRC cell lines or do untransformed FRCs also produce EVs. This question could certainly be addressed by electron scanning microscopy of freshly explanted FRCs.

2. In the previous publication from this group, the authors conclude that DCs are absolutely required for induction of Tregs in vivo and that a cooperation between DCs and stroll cells is needed for optimal induction of Treg.

3. More importantly, in the previous report, using a very similar culture system with unseparated stomal cells the authors conclude that production of TGFb plays little role in the inducing capacity of gut stromal cells from skin stromal cells as the superior inducing capacity of the gut stromal cells in maintained even in the presence of added TGFb.

4. The authors report the results for the presence of activated TGFb in their Elisa assay which is performed with acid activated supernatants. Physiologically, it is likely that latent TGFb is secreted by the stromal cells and must then be activated to exert biological activity. What activates the TGFb in their system? In general, TGFb is activated by intern avb8. Do the transformed FRC lines express intern b8? b8 is not expressed on Foxp3- T cells. The authors previously reported that mLN stromal cells express higher levels of b8 than skin stromal cells. In the absence of data on how TGFb is processed in their in vitro model, the significance of enhanced levels of TGFb in supernatants are dubious.

5. Lastly, one wonders if the data on the cell lines is even representative of FRCs in general as the authors state in the methods that "mLN-FRC cell line possessing the highest Treg induced capacity ......was chosen for further studies." This would appear to bias the conclusions of this report.

Reviewer: 2

Comments to the Author

Pasztoi and colleges have analyzed the tolerogenic potential of LN-derived FRC cell lines. They have identified that FRC cell line generated from mesenteric LN foster Treg induction via TGF-β containing microvesicles. This is a novel cellular communication within the lymph node between the stromal cell compartment and CD4 T cells.

The study is very well done, I have just minor comments.

1. Supplementary figure legend: please state what LD and CTV is.
2. Do iFRC cell line derived from sLN also able to produce MVs? It could be added to the discussion.

European Journal of Immunology

Reviewer: 3

Comments to the Author

The manuscript by Pasztoi et al presents data explaining mechanisms by which Treg induction is enhanced in gut-draining mesenteric lymph nodes (mLN) versus peripheral lymph nodes (pLN), which is proposed to be an important mechanism by which self-harmful inflammatory responses are controlled in the intestine. Specifically, the authors propose that extracellular vesicles derived from mLN fibroblastic reticular cells (FRCs), but not pLN FRCs carry TGFβ that promotes Treg induction. Suh a finding would be an important insight into new mechanisms promoting immune tolerance in the intestine. However, more characterisation of extracellular vesicles is required to conclude that they are responsible for presented findings, that the immortalised cell system used is indicative of the native untransformed cells, and also more explanation/discussion of the current results in the context of previous results from the same group that suggest TGFβ is not involved in preferencial Treg induction by mLN FRCs (reference 12 in the submitted manuscript.

Major comments

1. The characterisation of the microvesicles is limited, with only EM used to determine that vesicles have been purified. As suggested in position papers (e.g. https://www.ncbi.nlm.nih.gov/pmc/articles/PMC4275645/) attempts should be made to better characterise the vesicles by proteins they express to confirm that the preparations are truly extracellular vesicles and not contaminated with other membrane compartments.

2. The immortalised FRC cell lines are a useful tool for obtaining increased numbers of cells for study, but conclusions reached would be strengthened if authors could demonstrate that non-transformed FRCs produce the microvesicles with Treg-inducing ability. It is appreciated that obtaining sufficient cell numbers may be an issue for non-transformed cells, but could you confirm with supernatant from non-transformed cells?

3. A previous paper published by this group (reference 12 in current manuscript) has indicated that TGFβ is not important in the enhanced ability of mLN stromal cells to induce Tregs versus pLN stromal cells, as addition of active TGFβ does not rescue the Treg-inducing ability of pLN stromal cells to that of mLN stromal cells (Fig 3C of reference 12). This finidng is in contrast to the conclusions reached in the current manuscript that mLN stromal cells promote enhanced Treg induction via TGFβ-dependent mechanisms. An explanation for these opposite results should be proposed, as at present discussion of this discrepancy is lacking.

4. It is unclear to me whether the authors think that the TGFβ is on the outside or inside of the microvesicles. What are the methods for the ELISA they use? Does this involve lysis of the vesicles to release intravesicle TGFβ? Or is all the TGFβ measured stuck to the outside of the vesicle? More explanation/discussion in this direction is required.

Minor comments

1. The PDF at present is enormously long (2945 pages) dues to inclusion of RNA-seq data in the PDF (which cannot be interpreted due to being spread over many pages and words hidden In columns). This needs attaching as a supplementary table in Excel format rather than attached to a PDF.

2. In figure 3C, there appears to be no significant difference between no treatment and α-TGFβ antibody treated mLN-FRC SN treated cells? Is this the case as with pair-wise analysis it appears that there should be a significant difference? If not significant, this needs to be clearly ointed out in the text.

3. In figure 4B, there are black arrows pointing to structures, but no description as to what these arrows are in the figure legend.

First Revision – authors' response:

12-Jun-2017

**Reviewer # 1 comments**	**Response by the authors**
Concern #1:<br>This paper only describes the genetic and functional properties of a limited number of cells lines generated in vitro from fibroblastic reticular cells from mesenteric LN versus skin draining LN.<br>1. It is unclear if the properties of these cells lines actualy are similar to the properties of FRCs in vivo or freshly explanted in vitro. For example, the authors demonstrate that the lines produce extracellular vesicles containing TGF-b. Is this a property of transformed FRC cell lines or do untransformed FRCs also produce EVs. This question could certainly be addressed by electron scanning microscopy of freshly	We thank the reviewer for this comment. According to the literature, there are plenty of studies describing that the release of EVs is a property of almost all cell types under steady-state conditions, which can be increased during activation, cell death or under inflammatory circumstances (Szajnik, *PLoS One*, 2010, 7:e11469; György, *Cell Mol Life Sci*, 2011, 68:2667-88; Okoye, *Immunity*, 2014, 41:89-103; Yáñez-Mó, *J Extracell Vesicles*, 2015, 4:2706). Moreover, the capacity of different fibroblast types to produce EVs is also well known (Pasztoi, *Immunol Lett*, 2013, 152:25-31; Zou, *Hum Gene Ther*, 2016, 27:926-935; Van Pham, *Cytotechnology*, 2017, doi: 10.1007/s10616-017-0095-2), and many cells were shown to release EVs with suppressive capacity

explanted FRCs.	(Dubrot, *J Exp Med*, 2014, 211:1153-1166; Shen, *Eur J Immunol*, 2017, 47:900-910). Therefore, it is expected that the release of EVs is not a unique property of iFRCs, but *ex vivo* FRCs can also produce them. To prove this, we performed scanning electron microscopy on both mLN- and pLN-iFRCs, as well as on *ex vivo* $CD45^{-}CD24^{-}gp38^{+}CD31^{-}$ sorted FRCs. Indeed, the pictures demonstrated the capacity of all tested cell types to release EVs at a comparable level. We did not detect differences dependent on their original location (mLN compared to pLN) or cell type (*ex vivo* compared to immortalized cell lines). We have bundled these findings into the Figure 4 and novel Supplementary Figure 3, and have added corresponding sentences to “Introduction” (page 4), "Materials and Methods" (page 17), "Results" (page 8), “Discussion” (pages 12-13) and "Figure Legends" (page 28) in the revised version of the manuscript.
Concern #2:<br>In the previous publication from this group, the authors conclude that DCs are absolutely required for induction of Tregs in vivo and that a cooperation between DCs and stroll cells is needed for optimal induction of Treg.	We thank the reviewer for pinpointing this very interesting connection between the two studies. Indeed, it was shown in the previous publication of our group (Cording, *Mucosal Immunol*, 2014, 7:359-368) that DCs are critically required for *in vivo* Treg induction. However, it was also shown in this paper that under *in vitro* conditions *ex vivo* isolated FRCs alone - isolated from mLN and pLN - have a differential Treg-inducing capacity when cultured with naive $CD4^{+}$ T cells in the presence of CD3/CD28-coated beads, mimicking TCR activation by DCs. Since this previous finding was an interesting hint for a direct interaction between FRCs and T cells, we here set up an experimental system to better understand the mechanism of this crosstalk. In the revised version of the manuscript, we tried to emphasize this aspect better, and therefore we extended parts of the “Introduction” (page 4) and the "Discussion" (page 10).

Concern #3:<br><br>More importantly, in the previous report, using a very similar culture system with unseparated stomal cells the authors conclude that production of TGFb plays little role in the inducing capacity of gut stromal cells from skin stromal cells as the superior inducing capacity of the gut stromal cells in maintained even in the presence of added TGFb.	We agree with the reviewer that in our previous report (Cording, *Mucosal Immunol*, 2014, 7:359-368) the superior Treg-inducing capacity of mLN compared to pLN stromal cells was maintained after addition of exogenous TGF-β, but:<br><br>(i) This provides information about the need of stromal cells for additional TGF-β (which might come from DCs or other cells in their microenvironment), but does not explain the mechanism how FRCs directly induce Tregs, which was the focus of our manuscript.<br>(ii) The *in vitro* culture conditions in the previous paper included unsorted LN stromal cells. This means that besides FRCs, lymphatic endothelial cells (LECs), blood endothelial cells (BECs) or double negative (DN) stromal cells could be also present in the culture, which might result in a different outcome. However, due to the location of FRCs in the T cell zone of the LN (Bajénoff, *J Immunol*, 2008, 181:3947-54) these cells are likely the most relevant stromal cell subset in this context. Thus, we exclusively investigated the interaction between FRCs and T cells in the present study.<br>(iii) In the current manuscript we found a significantly higher TGF-β1 expression in mLN- compared to pLN-iFRCs that was also shown in a previous paper studying *ex vivo* FRCs (Malhotra, *Nat Immunol*, 2012, 13:499-510). Although this was not observed in the previous paper of the group (Cording, *Mucosal Immunol*, 2014, 7:359-368), the differential TGF-β1 expression was most probably blurred by the above-mentioned composition of diverse LN stromal cell subsets. In the revised version of this manuscript, we have clarified this point and have added further sentences and relevant references to the "Discussion" (pages 11-12).
Concern #4:<br><br>The authors report the results for the presence of activated TGFb in their Elisa assay which is performed with acid activated supernatants. Physiologically, it is likely that latent TGFb is secreted by the stromal cells and must then be activated to exert	We agree with the reviewer that TGF-β activation is a critical point to understand the mechanism of Treg-induction by FRCs. In the current literature, the integrin β8-mediated TGF-β activation by intestinal DCs (Worthington, *Gastroenterology*, 2011, 141:1802-12; Fenton, *Mucosal Immunol*, 2017,

biological activity. What activates the TGFb in their system? In general, TGFb is activated by intern avb8. Do the transformed FRC lines express intern b8? b8 is not expressed on Foxp3- T cells. The authors previously reported that mLN stromal cells express higher levels of b8 than skin stromal cells. In the absence of data on how TGFb is processed in their in vitro model, the significance of enhanced levels of TGFb in supernatants are dubious.	10:624-634) and Tregs (Worthington, *Immunity*, 2015, 42:903-15) is well described. Similarly, the increased expression of integrin αvβ8 on mLN stromal cells could contribute to their stably imprinted Treg-inducing capacity as discussed before (Cording, *Mucosal Immunol*, 2014, 7:359-368). Accordingly, the RNA-seq analysis on iFRCs revealed the expression of *Itgb8* at significantly higher levels in mLN-iFRCs as compared to pLN-iFRCs (log2FC -2.22, $p < 0.0001$). This observation is in line to what has been reported in our previous publication (Cording, *Mucosal Immunol*, 2014, 7:359-368), provides a possible mechanism of processing iFRC-derived TGF-β1 and argues for the similarities between iFRCs and *ex vivo* isolated stromal cells. We included these data as novel Supplementary Figure 2, and slightly expanded the corresponding parts of the “Results” and the "Discussion" sections with relevant references (pages 7, 13).
Concern #5:<br><br>Lastly, one wonders if the data on the cell lines is even representative of FRCs in general as the authors state in the methods that "mLN-FRC cell line possessing the highest Treg induced capacity ......was chosen for further studies." This would appear to bias the conclusions of this report.	We thank the reviewer for pinpointing this critical issue. Theoretically, the process of immortalization holds the risk of changing the cellular phenotype and generating gene expression differences. On the other hand, it is a valuable method to enable the analysis of cells available only at very limited numbers. Our motivation was to allow non-restricted investigations of stromal cells and to establish a DC-free Treg induction assay. Therefore, we generated three independent pairs of mLN- and pLN-iFRCs, and tested their Treg-inducing capacity. All three pLN-iFRCs had almost no Treg-inducing capacity, whereas two out of three mLN-iFRC cell lines harbored high Treg-inducing capacity, a finding corresponding to normal biological variance. Finally, we have chosen the pair of mLN- and pLN-iFRCs, which most closely resembled the differential Treg-inducing capacity of *ex vivo* isolated stromal cells (Cording, *Mucosal Immunol*, 2014, 7:359-368), where Treg-induction was statistically not different. We apologize for the imprecise description in the manuscript and have rephrased the corresponding part of the “Materials and Methods” (page 15).

Reviewer # 2 comments	Response by the authors
Minor concern #1:<br>Pasztoi and colleges have analyzed the tolerogenic potential of LN-derived FRC cell lines. They have identified that FRC cell line generated from mesenteric LN foster Treg induction via TGF-β containing microvesicles. This is a novel cellular communication within the lymph node between the stromal cell compartment and CD4 T cells.<br>The study is very well done, I have just minor comments.<br>1. Supplementary figure legend: please state what LD and CTV is.	We thank the reviewer for recognizing the inadequate definition of "LD" and "CTV" in the Legend of the Supplementary Figure 1. We have added the necessary information to the legend.
Minor concern #2:<br>Do iFRC cell line derived from sLN also able to produce MVs? It could be added to the discussion.	We thank the reviewer for this comment and agree that the EV-releasing capacity of pLN-iFRCs should also be discussed. According to the literature, there are plenty of studies describing that the release of EVs is a property of almost all cell types under steady-state conditions, which can be increased during activation, cell death or under inflammatory circumstances (Szajnik, *PLoS One*, 2010, 7:e11469; György, *Cell Mol Life Sci*, 2011, 68:2667-88; Okoye, *Immunity*, 2014, 41:89-103; Yáñez-Mó, *J Extracell Vesicles*, 2015, 4:2706). Moreover, the capacity of different fibroblast types to produce EVs is also well known (Pasztoi, *Immunol Lett*, 2013, 152:25-31; Zou, *Hum Gene Ther*, 2016, 27:926-935; Van Pham, *Cytotechnology*, 2017, doi: 10.1007/s10616-017-0095-2), and many cells were shown to release EVs with suppressive capacity (Dubrot, *J Exp Med*, 2014, 211:1153-1166; Shen, *Eur J Immunol*, 2017, 47:900-910). Therefore, it is expected that both mLN- and pLN-iFRCs release EVs, which we proved by scanning electron microscopy on both cell lines (and also on *ex vivo* $CD45^-CD24^-gp38^+CD31^-$ sorted FRCs). Moreover, we determined the size distribution with tunable resistive pulse sensing method, and the expression of EV-specific molecules by flow cytometry (see novel Supplementary Figure S3), and all novel findings argue for a similar capacity of mLN- and pLN-iFRCs to

	release EVs. We have implemented these findings into the Figure 4, novel Supplementary Table 2, and novel Supplementary Figure 3, and have added corresponding sentences to the “Introduction” (page 4), "Materials and Methods" (page 14, 17, 19), "Results" (page 8), “Discussion” (pages 12-13) and "Figure Legends" (page 28) in the revised version of the manuscript.

European Journal of Immunology

Reviewer # 3 comments	Response by the authors
Major concern #1:<br>The manuscript by Pasztoi et al presents data explaining mechanisms by which Treg induction is enhanced in gut-draining mesenteric lymph nodes (mLN) versus peripheral lymph nodes (pLN), which is proposed to be an important mechanism by which self-harmful inflammatory responses are controlled in the intestine. Specifically, the authors propose that extracellular vesicles derived from mLN fibroblastic reticular cells (FRCs), but not pLN FRCs carry TGFβ that promotes Treg induction. Suh a finding would be an important insight into new mechanisms promoting immune tolerance in the intestine. However, more characterisation of extracellular vesicles is required to conclude that they are responsible for presented findings, that the immortalised cell system used is indicative of the native untransformed cells, and also more explanation/discussion of the current results in the context of previous results from the same group that suggest TGFβ is not involved in preferencial Treg induction by mLN FRCs (reference 12 in the submitted manuscript.<br>Major comment 1. The characterisation of the microvesicles is limited, with only EM used to determine that vesicles have been purified. As suggested in position papers (PMC4275645) attempts should be made to better characterise the vesicles by proteins they express to confirm that the preparations are truly extracellular vesicles and not contaminated with other membrane compartments.	We thank the reviewer for this comment and agree that a more thorough characterization of iFRC-derived vesicles can be done. Although the generation of iFRC cell lines enabled the analysis of their Treg-inducing capacity, experiments with iFRC-derived EVs can be only performed in a quite limited number, due to the excessive need for pure fibronectin and high cell numbers. Therefore, in the first version of the manuscript we provided scanning electron microscopy and TEM pictures only to prove the EV-releasing capacity of mLN-iFRCs, the presence of MVs in the isolated pellet of mLN-iFRC-derived SN, and showing the structure of EVs surrounded by lipid bilayer (Figure 4). In the revised version of the manuscript, we extended the characterization of iFRC-derived EV samples:<br>(i) We aimed to show that the release of EVs is not a unique property of iFRCs, but *ex vivo* FRCs can also produce them. To prove this, we performed scanning electron microscopy on both mLN- and pLN-iFRCs, as well as on *ex vivo* $CD45^{-}CD24^{-}gp38^{+}CD31^{-}$ sorted FRCs. The pictures indeed demonstrated the capacity of all tested cell types to release EVs at comparable levels, which was not dependent on their original location (mLN compared to pLN) or cell type (*ex vivo* versus immortalized cell lines). We extended the original Figure 4 and generated a novel Supplementary Figure 3A with these data, and added corresponding sentences to "Results" (page 8), "Discussion" (pages 12-13), "Materials and Methods" (page 17), and "Figure Legends" (page 28) in the revised version of the manuscript.<br>(ii) We determined the size distribution of mLN- and pLN-iFRC-derived MV samples with tunable resistive pulse sensing (TRPS) analysis, and observed a similar size distribution of EVs in both samples, which corresponds to the expected size range of MVs (100-1000nm). These data are included into the novel Supplementary Table 2 and Supplementary Figure 3B, and corresponding sentences have been added to the "Results" (page 8) and "Materials and Methods" (page 19) sections in the revised version of the manuscript.<br>(iii) We characterized the expression of several EV markers (Annexin V, CD9, CD63) (Lötvall, *J Extracell Vesicles*, 2014, 3:10) and molecules of the donor cell

	(gp38, CD40) (Nakayama, *Transplantation*, 2015, 99:1561-7; Fuhrmann, *J Control Release*, 2015, 205:35-44) on mLN- and pLN-iFRC-derived MV samples by flow cytometry, which revealed the expression of EV markers on both samples, and showed no obvious difference between mLN- and pLN-iFRC-derived MVs. These findings are summarized in the novel Supplementary Figure 3C, and corresponding sentences have been added to the "Results" (page 8) and "Materials and Methods" (pages 14, 19) sections in the revised version of the manuscript.
Major concern #2:<br><br>The immortalised FRC cell lines are a useful tool for obtaining increased numbers of cells for study, but conclusions reached would be strengthened if authors could demonstrate that non-transformed FRCs produce the microvesicles with Treg-inducing ability. It is appreciated that obtaining sufficient cell numbers may be an issue for non-transformed cells, but could you confirm with supernatant from non-transformed cells?	We thank the reviewer for raising this question. We tried to culture *ex vivo* $CD45^{-}CD24^{-}gp38^{+}CD31^{-}$ sorted FRCs to produce sufficient amount of supernatant for the mentioned *in vitro* Treg-induction assay. Unfortunately, this kind of experiment had several drawbacks, which did not allow to fully answer the question raised. The most critical reasons include the very high number of mice required for the experiment, and the *in vitro* culture conditions, which - mainly due to the low cell numbers - do not support stromal cell viability. However, we could isolate enough cells for scanning electron microscopy in order to show that the release of EVs is not a unique property of iFRCs, but *ex vivo* FRCs can also produce them (Figure 4 and novel Supplementary Figure 3A), and it is not affected by the procedure of immortalization. Although our observation is based on experiments with immortalized cells, it is tempting to speculate that similar to mLN-iFRCs *ex vivo* FRCs from mLN can contribute to a high Treg-induction.
Major concern #3:<br><br>A previous paper published by this group (reference 12 in current manuscript) has indicated that TGFβ is not important in the enhanced ability of mLN stromal cells to induce Tregs versus pLN stromal cells, as addition of active TGFβ does not rescue the Treg-inducing ability of pLN stromal cells to that of mLN stromal cells (Fig 3C of reference 12). This finidng is in contrast to the conclusions reached in the current manuscript that mLN stromal cells promote	We agree with the reviewer that in the previous report (Cording, *Mucosal Immunol*, 2014, 7:359-368) the superior Treg-inducing capacity of mLN compared to pLN stromal cells was maintained after addition of exogenous TGF-β.<br>(i) However, it provides information about the need of stromal cells for additional TGF-β (which might come from DCs or other cells in their microenvironment), but does not fully explain the mechanism how FRCs directly induce Tregs, which was the focus of our manuscript.<br>(ii) On the other hand, the *in vitro* culture conditions

enhanced Treg induction via TGF β -dependent mechanisms. An explanation for these opposite results should be proposed, as at present discussion of this discrepancy is lacking.	in the previous paper included unsorted LN stromal cells. This means that besides FRCs, lymphatic endothelial cells (LECs), blood endothelial cells (BECs) or double negative (DN) stromal cells could be also present in the culture, which might result in different outcome. However, due to the location of FRCs in the T cell zone within the LN (Bajénoff, *J Immunol*, 2008, 181:3947-54), these cells are likely the most relevant stromal cell subset in this context. Thus, we exclusively investigated the interaction between FRCs and T cells in the present study.<br>(iii) Additionally, in the current manuscript we found a significantly higher TGF-β1 expression in mLN- compared to pLN-iFRCs that was also shown in a previous paper studying *ex vivo* FRCs (Malhotra, *Nat Immunol*, 2012, 13:499-510). Although this was not observed in the previous paper of our group (Cording, *Mucosal Immunol*, 2014, 7:359-368), the differential TGF-β1 expression was most probably blurred by the above-mentioned composition of diverse LN stromal cell subsets. In the revised version of this manuscript, we have clarified this point and have added further sentences and relevant references to the "Discussion" (pages 11-12).<br>(iv) In the previous paper an increased *Itgb8* expression of mLN stromal cells was reported (Cording, *Mucosal Immunol*, 2014, 7:359 368), which could be confirmed by RNA-seq analysis for mLN-iFRCs in the current study (log2FC -2.22, $p < 0.0001$, novel Supplementary Figure 2). This observation provides a possible mechanism of processing iFRC-derived TGF-β1 and argues for similarities between iFRCs and *ex vivo* stromal cells. We included these data as novel Supplementary Figure 2 and expanded the corresponding parts in the text (pages 7, 13).<br>(v) Finally, we think that the enhanced Treg-inducing capacity of mLN-iFRCs via TGF-β-carrying vesicles is not the only, but one of the major mechanisms responsible for peripheral tolerance, which can also explain the differences between the two studies. Accordingly, we have corrected the misleading sentence in the "Introduction" section (page 4).
Major concern #4:<br>It is unclear to me whether the authors think that the TGFβ is on the outside or inside of the microvesicles. What are the methods for the ELISA they use? Does this involve lysis of the vesicles to release intravesicle TGFβ?	We thank the reviewer for pinpointing this interesting detail regarding the location of vesicular TGF-β. The ELISA assay that was used to determine TGF-β concentration includes addition of acid to activate TGF-β. During this step, it is highly probable that vesicles are disrupted in the acidic environment, and

Or is all the TGFβ measured stuck to the outside of the vesicle? More explanation/discussion in this direction is required.	their TGF-β content was released. To our knowledge, there are no ELISA kits available using other TGF-β activation steps, which do not lead to membrane rupture. Moreover, due to the storage conditions, the EV samples underwent a freeze-thawing cycle prior to the ELISA assay, which could also disrupt their membrane integrity and makes latent TGF-β accessible for activation. Consequently, the performed assay does not allow discriminating between surface-bound and intravesicular TGF-β. We have clarified this point in the “Results” (page 9).
Minor concern #1:<br>The PDF at present is enormously long (2945 pages) dues to inclusion of RNA-seq data in the PDF (which cannot be interpreted due to being spread over many pages and words hidden In columns). This needs attaching as a supplementary table in Excel format rather than attached to a PDF.	We agree with the reviewer that the PDF automatically generated in the electronic submission system was too long. During the submission procedure we have immediately contacted the Editorial Office. We also asked for help to correct the submitted manuscript and to send the reviewers a PDF without Supplementary Table 1. We apologize for this and hope that for the resubmission this problem can be solved.
Minor concern #2:<br>In figure 3C, there appears to be no significant difference between no treatment and α-TGFβ antibody treated mLN-FRC SN treated cells? Is this the case as with pair-wise analysis it appears that there should be a significant difference? If not significant, this needs to be clearly ointed out in the text.	We thank the reviewer this comment. We performed one-way ANOVA followed by Bonferroni`s post-test to analyze these data, but Treg induction between treated (anti-TGF-β) and untreated samples (mLN-iFRC or mLN-iFRC SN) was statistically not significant, as shown in the Figure accordingly.
Minor concern #3:<br>In figure 4B, there are black arrows pointing to structures, but no description as to what these arrows are in the figure legend.	We apologize for the imprecise explanation of Figure 4. The black arrows indicate the lipid bilayer of the depicted vesicles, which we corrected in the corresponding part of the “Figure Legend” (page 28).

Second Editorial Decision

30-Jun-2017

Dear Prof. Huehn,

Thank you for submitting your revised manuscript ID eji.201746960.R1 entitled " Mesenteric lymph node stromal cell derived extracellular vesicles contribute to peripheral de novo induction of Foxp3+ regulatory T cells" to the European Journal of Immunology. Your manuscript has been re-reviewed and the

comments of the referees are included at the bottom of this letter.

Although the referees have recommended publication, some revisions to your manuscript have been requested. Therefore, we invite you to respond to the comments of the referees and revise your manuscript accordingly. In particular, we encourage you to provide answers to Referee 1 and discuss the limitations of the study. For transparency, it would be important to include discussion of these limitations to your revised manuscript (discussion section).

You should also pay close attention to the editorial comments included below. **In particular, please edit your figure legends to follow Journal standards as outlined in the editorial comments. Failure to do this will result in delays in the re-review process.**

If the revision of the paper is expected to take more than three months, please inform the editorial office. Revisions taking longer than six months may be assessed by new referee(s) to ensure the relevance and timeliness of the data.

Once again, thank you for submitting your manuscript to European Journal of Immunology. We look forward to receiving your revision.

Yours sincerely,
Nadja Bakocevic

on behalf of
Prof. Shimon Sakaguchi

Dr. Nadja Bakocevic
Editorial Office
European Journal of Immunology
e-mail: ejied@wiley.com

www.eji-journal.eu

Reviewer: 1

Comments to the Author

1. The authors have addressed many of my technical criticisms. However, the in vivo relevance of the findings remains unclear as they could not obtain sufficient numbers of freshly explanted cells to test whether similar results would occur with non-transformed cells. This is a major limitation of this paper.
2. Although the authors clearly demonstrate that the cell lines express Itgb8, it is not clear if the EVs express this intern. If not, how is the TGFb present in the ENs processed in the reductionist protocol used in this manuscript. No other cells except ENs and responder T cells are present in the culture.
3. Only small percentages of Foxp3+ T cells are induced. Is this a biologically significant finding?

Reviewer: 3

Comments to the Author
The authors have done a good job of addressing the points raised in my original review. A few minor points remain:
- In both Figure 3 (B and C), as confirmed by the authors, the addition of a TGFÎ²-blocking antibody does not significantly inhibit Treg induction by mLN FRC cells or s/n. Although it is appreciated that there is a strong trend, likely insufficient experiments have been performed to achieve statistical significance. It is also appreciated that there is no significant difference between medium treated and anti-TGFb antibody treated samples, and a significant difference between non-treated and mLN FRC cell/sn-treated in support of a role for TGF-beta, and also that MV data later In the manuscript support a role for TGF. However, coupled with the fact that previous publications from the same authors do not support a role for TGFb in stromal cell induction of Tregs, I feel it is pertinent to clearly state that there is a non-significant trend in figure 3 rather than the definitive statements at present (so readers are better informed to make up their own minds as to the likelihood of importance of TGFb).
- For RNA-seq data for itgb8, is the p value cited an adjusted p-value? Adjusted p values are crucial to correct for multiple comparisons when comparing thousands of genes in RNA-seq data. This needs to be cler, and, if the p-adjusted value is not significant, the data needs to be re-assessed and the conclusions drawn about itgb8 levels removed. If the p value given is a p-adjusted value, this should be more clearly stated.
- The grey isotype control plots in histograms of supplementary figure 3 should be re-coloured as they can´t be seen at all clearly.

Second Revision – authors' response:

12-Jul-2017

Reviewer # 1 comments	Response by the authors

Concern #1:<br><br>The authors have addressed many of my technical criticisms. However, the in vivo relevance of the findings remains unclear as they could not obtain sufficient numbers of freshly explanted cells to test whether similar results would occur with non-transformed cells. This is a major limitation of this paper.	We thank the reviewer for this comment. For better transparency, we have addressed this issue in the "Discussion" section (page 10) of the manuscript.
Concern #2:<br><br>Although the authors clearly demonstrate that the cell lines express Itgb8, it is not clear if the EVs express this intern. If not, how is the TGFb present in the ENs processed in the reductionist protocol used in this manuscript. No other cells except ENs and responder T cells are present in the culture.	We thank the reviewer for pinpointing this very interesting detail of TGF-β activation. Indeed, we do not have data about the presence of on iFRC-derived EVs. However, it is well known that EVs carry molecules of donor origin (Gyorgy et al., *Cell Mol Life Sci*, 2011, 68:2667-2688), which supports the hypothesis mentioned in the manuscript that integrin β_8 on EVs might be responsible for TGF-β activation. It is also possible that readily differentiated Tregs represent a second line of TGF-β activation due to the expression of *Itgb8* (Worthington et al., *Immunity*, 2015, 42:903-915) as discussed in the paper. Furthermore, EVs are well known carriers of biologically active enzymes (Pasztoi et al., *Immunol Lett,* 2013, 149:71-76), thus they might carry matrix metalloproteinases involved in TGF-β activation, such as MMP-2 and -9 (Yu et al., *Genes Dev*, 2000, 14:163-176; Annes et al., *J Cell Sci*, 2003, 116:217-224), which are both expressed on pLN- and mLN-iFRCs (Supplementary Table 1). In the second revision of the manuscript, we extended this part of the "Discussion" section with the first explanations only for the sake of clarity (page 13).

Concern #3:<br>Only small percentages of Foxp3+ T cells are induced. Is this a biologically significant finding?	We thank the reviewer for this comment. Our *in vitro* cultures show a comparable level of Treg induction as reported previously by others (Esterhazy et al., *Nat Immunol*, 2016, 17:545-555; Coombes et al., *J Exp Med*, 2007, 204:1757-1764). Although Treg induction by mLN-iFRC-derived MVs reached a low level of 3-4 % only, we observed a dose-dependent increase in Treg induction upon increasing the amount of mLN-iFRC MVs added to the cultures, and could reach the frequency of Tregs induced by mLN-iFRCs or mLN-iFRC SN (data not shown). Taken together, we think that the level of Treg induction in our *in vitro* system is comparable to what has been reported in the abovementioned studies.

**Reviewer # 3 comments**	**Response by the authors**
Minor concern #1:<br>In both Figure 3 (B and C), as confirmed by the authors, the addition of a TGFβ-blocking antibody does not significantly inhibit Treg induction by mLN FRC cells or s/n. Although it is appreciated that there is a strong trend, likely insufficient experiments have been performed to achieve statistical significance. It is also appreciated that there is no significant difference between medium treated and anti-TGFb antibody treated samples, and a significant difference between non-treated and mLN FRC cell/sn-treated in support of a role for TGF-beta, and also that MV data later In the manuscript support a role for TGF. However, coupled with the fact that previous publications from the same authors do not support a role for TGFb in stromal cell induction of Tregs, I feel it is pertinent to clearly state that there is a non-significant trend in figure 3 rather than the definitive statements at present (so readers are better informed to make up their own minds as to the likelihood of importance of TGFb).	We thank the reviewer for recognizing the misleading description of our findings shown in Figure 3. We rephrased the corresponding sentences accordingly in the “Results” section (page 7).

Minor concern #2:<br><br>For RNA-seq data for itgb8, is the p value cited an adjusted p-value? Adjusted p values are crucial to correct for multiple comparisons when comparing thousands of genes in RNA-seq data. This needs to be cler, and, if the p-adjusted value is not significant, the data needs to be re-assessed and the conclusions drawn about itgb8 levels removed. If the p value given is a p-adjusted value, this should be more clearly stated.	We thank the reviewer for this comment and apologize for the incorrect description of the corresponding p-values. The p-values of the Itgb8 expression data were adjusted p-values, consequently we have corrected it in the "Results" section (page 7).
Minor concern #3:<br><br>The grey isotype control plots in histograms of supplementary figure 3 should be re-coloured as they can't be seen at all clearly.	We agree with the reviewer that the colours of the Supplementary Figure 3 could be improved. We have changed Supplementary Figure 3 accordingly.

Third Editorial Decision

18-Jul-2017

Dear Prof. Huehn,

It is a pleasure to provisionally accept your manuscript entitled " Mesenteric lymph node stromal cell derived extracellular vesicles contribute to peripheral de novo induction of Foxp3+ regulatory T cells" for publication in the European Journal of Immunology. For final acceptance, please follow the instructions below and return the requested items as soon as possible as we cannot process your manuscript further until all items listed below are dealt with.

Please note that EJI articles are now published online a few days after final acceptance (see Accepted Articles: http://onlinelibrary.wiley.com/journal/10.1002/(ISSN)1521-4141/accepted). The files used for the Accepted Articles are the final files and information supplied by you in Manuscript Central. You should therefore check that all the information (including author names) is correct as changes will NOT be permitted until the proofs stage.

We look forward to hearing from you and thank you for submitting your manuscript to the European Journal of Immunology.

European Journal of Immunology

Yours sincerely,
Nadja Bakocevic

on behalf of
Prof. Shimon Sakaguchi

Dr. Nadja Bakocevic
Editorial Office
European Journal of Immunology
e-mail: ejied@wiley.com
www.eji-journal.eu
